# Effectiveness and harms of clinical decision support systems for referral within chronic pain practice: protocol for a systematic review and meta-analysis

**DOI:** 10.1186/s13643-021-01596-7

**Published:** 2021-02-09

**Authors:** Hervé Tchala Vignon Zomahoun, Regina Visca, Nicole George, Sara Ahmed

**Affiliations:** 1 Faculty of Medicine, School of Physical and Occupational Therapy, Epidemiology, Biostatistics, and Occupational Health, McGill University, Montreal, Quebec Canada; 2 McGill University Health Centre, Montreal, Quebec Canada; 3 Department of Social and Preventive Medicine, Université Laval, Quebec, Quebec Canada; 4 VITAM Research Centre of Health Sustainability, Quebec, Quebec Canada; 5 McGill RUIS Centre of Expertise in Chronic Pain, Montreal, Quebec Canada; 6 Department of Family Medicine, McGill University, Montreal, Quebec Canada; 7 Centre de recherche interdisciplinaire en réadaptation (CRIR), Constance Lethbridge Rehabilitation Center du CIUSSS du Centre-Ouest-de-l’Île-de-Montréal, Montreal, Canada

**Keywords:** Clinical decision support, Referral, Chronic pain, Systematic review, Meta-analysis

## Abstract

**Background:**

Chronic pain is a common public health problem with negative consequences for individuals and societies. Fortunately, interdisciplinary chronic pain management has been shown to be effective for improving patients’ outcomes and strongly recommended in clinical practice guidelines. Appropriate referral within the healthcare system based on individuals’ needs and available services is essential to optimise health-related outcomes and maximise resources. Clinical decision support systems have been shown to be effective for supporting healthcare professionals in different practices. However, there is no knowledge synthesis on clinical decision support systems for referral within chronic pain practice. We aim to identify the clinical decision support systems for referral within chronic pain practices and assess their content, effectiveness, harms, and validation parameters.

**Methods:**

Using the methodology of Cochrane reviews, we will perform a systematic review and meta-analysis based on studies meeting the following criteria: *Population*, patients with chronic pain and/or healthcare professionals working in chronic pain; *Intervention*, clinical decision support systems for referral within chronic pain practice; *Comparison*, any other clinical tool, any usual care or practices; *Outcom*es, clinical outcomes of patients measuring how patients feel, function or survive including benefits, adverse effects, continuity of care, care appropriateness, care satisfaction, quality of life, healthcare professional performance, and cost outcomes; and *Study design*: randomized controlled trials, non-randomized controlled trials, before and after controlled studies and interrupted time series. We will search relevant literature with the support of an information specialist using Medline, Embase, PsycInfo, CINHAL, Web of Science and Cochrane Library from their inception onwards. Two reviewers will independently complete study selection, data extraction and risk of bias assessment. We will analyse data to perform both narrative syntheses and meta-analysis if appropriate.

**Discussion:**

Findings of this review will contribute to enhancing chronic pain care and research. Clinical decision support systems identified as effective in this review can be investigated for implementation in clinical practice and impact on improving patient, clinical and health system outcomes. Clinical decision support systems not yet ready for implementation that require further improvement will also be identified.

**Systematic review registration:**

PROSPERO registration: CRD42020158880.

**Supplementary Information:**

The online version contains supplementary material available at 10.1186/s13643-021-01596-7.

## Background

Pain is defined as an unpleasant sensory and emotional experience associated with, or resembling that associated with, actual or potential tissue damage [1]. Pain is frequently classified as chronic when it lasts three or more months beyond the time of healing [2]. Chronic pain is a common public health problem with negative consequences for individuals and societies. According to comprehensive literature reviews, the prevalence of chronic pain for general populations internationally ranged from 6 to 50% depending on the localization of pain, the age of patients and the country [3, 4]. In 2016, low back pain and migraine, which are among the most frequent forms of chronic pain, were two of the five main causes of years lived with disability contributing to 57.6 million and 45.1 million of total years lived with disability in worldwide, respectively [5]. Moreover, authors of a recent systematic review and meta-analysis showed that chronic pain is associated with cardiovascular diseases (pooled odds ratio of 1.73, 95% CI 1.42–2.04), with cerebrovascular diseases (pooled odds ratio of 1.81, 95% CI 1.51–2.10), and with mortality from cardiovascular diseases (pooled odds ratio of 1.20, 95% CI 1.05–1.36) [6].

Fortunately, there are treatments that effectively manage chronic pain and improve patients’ outcomes as shown in evidence-based clinical practice guidelines [7]. Recommendations from recent clinical guidelines based on high-quality evidence emphasize interdisciplinary management programs as being the best way to manage chronic pain [7]. More specifically, Oslund et al. have shown that interdisciplinary pain management programs improve and maintain patient outcomes across a range of domains (pain severity, interference, distress, control, helpfulness, and hours resting) within 1-year follow-ups [8]. Interdisciplinary pain management programs are recommended to better cover the complexity of chronic pain treatments that are multiple and categorized in two overarching approaches: (1) biomedical approach including pharmaceutical, surgical, and electrical stimulation treatments and (2) biopsychosocial approach including physiotherapy, occupational therapy, specialist nursing, clinical psychology, cognitive behavioural therapy, acceptance, and commitment therapy [9].

For chronic pain management to be effective, each interdisciplinary healthcare professional team member must have a clearly defined role with clear guidelines of when a patient should be referred to specialised providers and services. Appropriate referral decisions require consideration of a massive amount of information from patients, clinical assessments, and guidelines. Clinical decision support systems help to synthesise the information, thereby standardizing and facilitating the work of healthcare professionals. Clinical decision support systems are defined as being an application or software in which patient information is matched to clinical knowledge and patient-specific assessments or recommendations to help healthcare professionals or patients form a decision [10, 11]. According to the taxonomy by Wright et al., one distinguishes six types of clinical decision support systems including medication dosing support, order facilitators, point-of-care alerts/reminders, relevant information display, expert systems and workflow support [12].

In the literature, many clinical decision support systems were identified [13–15] with evidence supporting their effectiveness [14, 15]. For example, in a systematic review by Jenkins et al. [15], the effectiveness of intervention components including clinical decision support systems, targeted reminders, audits and feedback, practitioner education, and guideline dissemination for optimising use of imaging for low back pain was examined. Clinical decision support systems have been found effective for reducing the use of imaging for low back pain (absolute difference = 36.8%, 95% CI 33.2–40.5%) [15]. Although to a smaller extent compared to clinical decision support systems, targeted reminders were also found to be effective at reducing the use of imaging (absolute proportion difference = 22.5%, 95% CI 8.4–36.8%) [15]. In contrast, there was no evidence or sufficient data to determine the effectiveness of other components [15].

While individual studies have highlighted components of clinical decision support systems, to date, there is no knowledge synthesis on clinical decision support systems for referral between healthcare professionals or within chronic pain care continuums. A synthesis of content, supporting evidence for their validity, and the effectiveness of chronic pain referral decision support tools can substantially facilitate healthcare professionals’ decisions to refer individuals to services they need. We will perform a systematic review and meta-analysis to identify clinical decision support systems for referral to services or programs within chronic pain practices, and to assess their effectiveness and potential harms on patients, the quality of care, healthcare professional outcomes, economic outcomes and the evidence supporting their validity.

## Methods

This review is registered under the number CRD42020158880 on PROSPERO which is an International prospective register of systematic reviews [16]. We follow the Preferred Reporting Items for Systematic Reviews and Meta-Analyses-Protocols (PRISMA-P) review guidelines to draft our systematic review protocol (see the PRISMA-P checklist in Appendix file 1) [17]. The methodology presented is based on the Cochrane systematic review methods [18].

### Eligibility criteria

We defined the eligibility criteria of studies according to the PICOS approach (P = population, I = intervention, C = comparison, O = outcomes, S = study design) (see Appendix file 2, Table 1).

#### Population

We will consider studies based on patients with chronic pain or the ones in which a subgroup analysis was performed on patients with chronic pain. Pain is classified as chronic when it lasts three or more months beyond the time of healing. We will also include the studies in which participants are healthcare professionals who manage chronic pain patients.

#### Intervention

We will include studies in which the clinical decision support system for referral within chronic pain practices was explored. This clinical decision support system could have been validated or not. It could also have been used alone or with another intervention component. Clinical decision support system is defined as computer-based information systems used to integrate clinical and patient information and provide support for decision-making in patient care [19]; or mathematical or statistical procedures used as aids in making a decision [20]. They are frequently used in medical decision-making.

#### Comparison

Except the case of interrupted time series, we will consider for inclusion comparison groups such as the variants of clinical decision support systems, usual care and usual practice for referral in chronic pain.

#### Outcomes

We will consider studies assessing the following outcomes: (1) clinical outcomes of patients evaluating how they feel, function or survive including clinical decision support system benefits, adverse events or harms; and the patient perceptions about continuity of care, care appropriateness, care satisfaction and quality of life; (2) healthcare professional outcomes including, but not limited to, performance, workload and work satisfaction; and (3) economic outcomes including full and partial economic evaluation outcomes.

#### Study design

We will consider randomized controlled trials, non-randomized controlled trials, before and after controlled studies, interrupted time series with three point measures before and after the administration of clinical decision support system tested. Protocols, cross-sectional studies, case-control studies, pre-post or before-after studies without control group, and cohort studies will be excluded.

### Information source and search strategies

We will perform the literature search in three steps. First, an information specialist will develop the literature search strategy in MEDLINE (see preliminary literature search for MEDLINE in Appendix file 2, Table 2) and translate it, after validation and revision, in other bibliographic databases including Embase, PsycInfo, CINHAL, Web of Science, and Cochrane Library from their inception onwards. Second, the initial search strategy will be discussed and validated with the authors of the present review. Third, a second information specialist will revise the initial search strategy with the *Peer Review of Electronic Search Strategies* (*PRESS*) Tool [21]. We will use the following main concepts for the literature search: concepts related to clinical referral, the ones related to decision support and to pain. As we will consider only published studies, the grey literature will not be searched. Language and year of publication restrictions will not be applied.

### Study records

#### Data management

The list of references generated from each of the six databases will be imported to EndNote software. These will be merged and the reference duplicates between databases identified, and either automatically or manually removed. The list of unique references will be exported to an Excel file. The success of citations’ exportation will be ensured by a comparison of the number of references in the EndNote file to the one in the Excel file in order to verify that none of citations are missing.

#### Study selection process

We will perform the process of study selection in three steps. **Step 1**: Two reviewers (HTVZ, NG) will independently perform a pilot selection of studies on 10% of the total citations identified. This pilot aims to facilitate a shared understanding of eligibility criteria by reviewers and to clarify these criteria if needed for the study selection. **Step 2**: After a conclusive pilot step, reviewers will independently perform the selection of studies based on titles and abstracts. They will consider “include,” “exclude” or “unclear” as modalities of the studies selection. **Step 3**: All citations rated “include” or “unclear” will be considered for selection by full texts. This step of selection will also be rated “include”, “exclude” or “unclear”. Any disagreements between two reviewers will be discussed and resolved by consensus or else refereed by a third reviewer (SA) if needed. We will evaluate the magnitude of agreements between reviewers for the study selection using kappa statistics [22]. We will consider acceptable a kappa statistic greater than 0.60 for the pursuit of review process. If not, the eligibility criteria will be re-discussed, and the study selection will be redone. Corresponding or first authors of studies coded “unclear” will be contacted by email to obtain missing information or clarification needed.

#### Data collection process

Two reviewers (HTVZ, NG) will independently carry out data extraction using a data extraction form adapted from the guidance of data extraction by the Cochrane systematic review methodology [18]. We will consider the following variables regrouped in five categories: *study characteristics* including name of the first author, year of the publication, country of study population, settings and study design; *population characteristics* including total sample size, sex, age, nature of pain, socio-economic status, fragility and ethnicity; *characteristics of clinical decision support* systems including name, format, content, frequency of use, fidelity measure and decision support validation parameters; *characteristics of comparison group* including name, format, content, frequency of use, fidelity measure and decision support validation parameters of what was offered to participants of comparison groups; *outcome characteristics* including name of outcome, its scale, type of effect measure (e.g., odds ratio, relative risk, hazards ratio, mean difference), crude and adjusted effect measures, value of association measure and its 95% confidence interval. Reviewers will perform a conclusive data extraction pilot on two to three studies before the main data extraction. Any disagreements between reviewers will be discussed and resolved by consensus or else refereed by a third reviewer (SA) if needed.

#### Risk of bias in individual studies

Two reviewers will independently assess the risk of bias in included studies using the risk of bias assessment tool appropriate for the study design. For the randomized trials, we will use the revised Cochrane Risk-of-Bias Tool (RoB 2) [23]. This tool covers five bias domains including bias arising from the randomisation process, bias due to deviations from intended interventions, bias due to missing outcome data, bias in measurement of the outcome, and bias in selection of the reported result [23]. Each of these bias domains and overall risk-of-bias will be rated low risk-of-bias and high risk-of-bias, or in some concerns, we will use the Risk of Bias in Non-randomized Studies of Interventions (ROBINS-I) tool for quasi-experimental trials and interrupted time series trials [24]. This tool covers seven risk-of-bias domains including bias due to confounding, bias in selection of participants into the study, bias in classification of interventions, bias due to deviations from intended interventions, bias due to missing data, bias in measurement of outcomes, and bias in selection of the reported result [24]. Each of these bias domains and overall risk-of-bias will be rated no information, low, moderate or high risk-of-bias. The two reviewers (HTVZ, NG) will discuss disagreements. A third party (SA) will be consulted if there is no consensus between reviewers.

### Data synthesis

We will describe the process of study selection considering the total number of identified studies, the number of studies retained by title and abstract, and the number of studies retained after full-text screening. We will document the reasons for study exclusion according to the eligibility criteria using the PICOS elements. Data extracted will firstly be synthesized in narrative form considering the studies, populations, decision support and outcome characteristics. For each outcome of interest from randomized trials, we will synthesize the assessment of the risk-of-bias using a figure presenting the ratings for each of five bias domains and overall risk-of-bias. Low risk-of-bias will be coded with the green color, high risk-of-bias with the red color, and some concerns with yellow color on the figure of risk-of-bias assessment for each outcome. For each outcome of interest from non-randomized studies, we will also synthesize the assessment of the risk-of-bias using a figure presenting the ratings for each of seven bias domains and overall risk-of-bias. Low risk-of-bias will be coded with the green color, high risk-of-bias with the red color, moderate risk-of-bias with yellow color, and no information without color on the figure of risk-of-bias assessment for each outcome. Meta-analysis will be performed for each outcome of interest if sufficient and appropriate data is available in at least three studies retained. We will use odds ratio as effect size with its 95% confidence interval, and we will perform transformations of other types of effect size into odds ratio using the methodology proposed by Borenstein et al. [25]. Since we anticipate a potential heterogeneity between studies that will be included, we will use a random effect model to estimate the pooled effect size and its 95% confidence interval for each outcome of interest [25, 26]. We will quantify the heterogeneity with Higgins’ *I*^2^ (100% x (Q-df)/Q) that represents the percentage of variability between effect size estimates due to the heterogeneity rather than the chance (Higgins, 2002; Higgins, 2003). We will test it using the *Q* test [27, 28]. The values of *I*^2^ may be interpreted as follows: very low if < 25%; low if 25 to < 50%; moderate if 50 to < 75%; and high if 75% or more (Higgins, 2003) [28, 29]. We will explore the potential heterogeneity with subgroup analyses considering the following variables a priori: chronic pain definition (pain duration ≥ 3 months versus ≥ 6 months versus ≥ 12 months); nature of chronic pain management (multidisciplinary program or not); part of an integrated network (yes or no); types of decision support (e.g., expert systems versus workflow support); frequency of decision support use (< median of frequencies; ≥ median of frequencies); sex of participants (< median of women percentages; ≥ median of women percentages); economic level of participant countries (low-income versus middle-income versus high-income) and average age of participants (< median of average age; ≥ median of average age), and study design (RCTs versus non-randomised studies). The subgroup analyses will be done only in the case there are at least two studies in each subgroup. Finally, we will use the Bonferroni method to correct the *p* values obtained from multiple comparison tests based on the same studies [30]. Each of these *p* values will be multiplied by the number of comparison tests done to obtain the corrected *p* values determining the statistical difference between subgroups [30]. Moreover, we will evaluate the potential risk of publication bias using both the visual examination of funnel plots and the non-parametric “trim and fill” method [31]. This examination will be done when the number of studies included in the meta-analysis is equal to or greater than ten for each outcome of interest. Finally, we will perform sensitivity analyses excluding studies with high risk of bias from the pooled effect size estimation for each outcome of interest when applicable.

### Assessment of the quality of evidence

We will assess the quality of evidence for each outcome of interest with the Grading of Recommendations, Assessment, Development and Evaluation (GRADE) to reduce the potential misinterpretation of the findings in this review [32]. For each outcome of interest, we will initially rate overall certainty of evidence as low (+2) for the non-randomized studies, and high (+ 4) for the randomized trials [32]. We will downgrade the initial overall certainty using the following criteria: risk of bias, indirectness of evidence, data heterogeneity, imprecision of effect size estimates, and risk of publication bias [32]. The downgrading will be done considering the rating of each criterion as follows: no (0), probably no (− 0.5), probably yes (− 1) and yes (− 2) [32]. After that, we will upgrade (+1) the initial overall certainty of evidence using the following criteria: large intervention effect, dose-response effect and opposing plausible residual bias and confounding [32]. Therefore, the final overall certainty of evidence will be rated very low, low, moderate and high for each outcome of interest [32].

## Discussion

In this review, we aim to assess the available evidence on the description, support for the validity and effectiveness of the clinical decision support systems for referral in chronic pain. We will also classify the identified clinical decision support systems according to their level of effectiveness and the quality of existing evidence. The expected findings may have valuable practice implications for stakeholders including patients, health care professionals, health system managers or decision-makers, and researchers working in chronic pain. It is expected that our findings will inform stakeholders in chronic pain of existing and effective clinical decision supports for the appropriate referral of individuals across different chronic pain services. Our findings may also be used in practice guidelines to guide the appropriate referral of patients with chronic pain within health service networks. The use of our expected findings by healthcare professionals could contribute to making informed decisions about the choice of appropriate clinical decision support systems to use in order to enhance the process of care for individuals with chronic pain. For the research implications, our expected findings could generate relevant research questions related to decision making for effective chronic pain management. For example, clinical decision support systems identified as effective in this review could be investigated further to evaluate their implementation across various clinical practice settings, and their impact on patient, clinical and health system outcomes in real context. Moreover, clinical decision support systems not yet ready for implementation that require further adaptations will also be identified.

We plan to disseminate our findings to different audiences including patients with chronic pain, healthcare professionals, researchers and healthcare managers and health system decision-makers working in chronic pain. To optimize our dissemination activities, we will involve stakeholders including one healthcare professional who provides chronic pain care, two patient representatives with chronic pain and at least one researcher with experiences in chronic pain research. We will engage them as partners in the (1) data extraction and syntheses, (2) interpretation of our review’s findings, (3) choice and development of knowledge products and dissemination strategies, and (4) implementation of our dissemination activities. For each audience, we will adapt the content of messages drawn from our results and will identify champions for promoting and performing the dissemination activities. The dissemination of our activities will consist of publishing our protocol and review papers in a peer-reviewed journal, performing four oral or poster presentations at national and international conferences in the domain of chronic pain, diffusing our results in plain language on websites of the associations of chronic pain patients and healthcare professionals, and on media networks (e.g., Personal Facebook, Linkedin and Twitter of team members). For health system decision-makers, we will develop policy briefs to share our findings with them and engage them in an ongoing dialog about the practice implications of the findings. Moreover, we plan to perform two webinars to present our findings, one for healthcare professionals as a part of their continuing professional development activities, and the other for healthcare managers. We will evaluate our dissemination activities by considering participants’ perceptions about the relevance of content presented, their satisfaction level, their knowledge gain and the number and profiles of participants joined.

Since our research project is a systematic review and meta-analysis based on existing primary studies, it will not be necessary to ask for ethics approval. Representatives of potential end-users including healthcare professionals and patients with chronic pain will be considered as members of our team and will be compensated for their time and related costs (e.g., transport fees).

## Supplementary Information

**Additional file 1.** PRISMA-P 2015 Checklist.

**Additional file 2: Table 1.** Eligibility criteria for studies. **Table 2.** Preliminary search strategy in Ovid MEDLINE.

## Data Availability

We included in our manuscript all data and materials used at this step of our review. We do not have any additional data or materials at this step. However, we will state all data and materials used during the realisation of our review and they will be available from the corresponding author.

## Abbreviations

CINHAL: Cumulative Index to Nursing and Allied Health Literature
EMBASE: Excerpta Medica dataBASE
EPOC: Effective Practice and Organisation of Care
GRADE: Grading of Recommendations, Assessment, Development and Evaluation
MEDLINE: Medical Literature Analysis and Retrieval System Online
RCTs: Randomized controlled trials
PICOS: Population, Intervention, Comparison, Outcomes, Study design
PRISMA: Preferred Reporting Items for Systematic Reviews and Meta-Analyses
PRISMA-P: Preferred Reporting Items for Systematic Reviews and Meta-Analyses protocols
ROBINS-I: Risk of Bias in Non-Randomized Studies - of Interventions
RoB 2: A revised tool to assess risk of bias in randomized trials
