# Supplementary material for: Effectiveness and harms of clinical decision support systems for referral within chronic pain practice: protocol for a systematic review and meta-analysis
Source: Syst Rev. 2021 Feb 9;10:53. doi: 10.1186/s13643-021-01596-7 (PMC7874648; doi:10.1186/s13643-021-01596-7)
Supplement: Supplementary file 2 — Additional file 2: Table 1. Eligibility criteria for studies. Table 2. Preliminary search strategy in Ovid MEDLINE. [file 13643_2021_1596_MOESM2_ESM.docx]

**APPENDIX 2**

| **Table 1.:** Eligibility criteria for studies | |
| --- | --- |
| **PICOS elements** | **Eligibility criteria** |
| Population | We will consider studies based on patients with chronic pain or the ones in which a subgroup analysis was performed on patients with chronic pain. Pain is classified as chronic when it lasts three or more months beyond the time of healing. We will also include the studies in which participants are healthcare professionals who manage chronic pain patients. |
| Intervention | We will include studies in which the clinical decision support system for referral within chronic pain practices was explored. This clinical decision support system could have been validated or not. It could also have been used alone or with another intervention component. Clinical decision support system is defined as computer-based information systems used to integrate clinical and patient information and provide support for decision-making in patient care [19]; or mathematical or statistical procedures used as aids in making a decision [20]. They are frequently used in medical decision-making. |
| Comparison | Except the case of interrupted time series, we will consider for inclusion comparison groups such as the variants of clinical decision support systems, usual care, and usual practice for referral in chronic pain*.* |
| Outcomes | We will consider studies assessing the following outcomes: 1) clinical outcomes of patients evaluating how they feel, function, or survive including clinical decision support system benefits, adverse effects or harms; and the patient perceptions about continuity of care, care appropriateness, care satisfaction, and quality of life; 2) healthcare professional outcomes including, but not limited to, performance, workload, and work satisfaction; and 3) economic outcomes including full and partial economic evaluation outcomes. |
| Study designs | We will consider randomized controlled trials, non-randomized controlled trials, before and after controlled studies, interrupted time series with three point measures before and after the administration of clinical decision support system tested. Protocols, cross-sectional studies, case-control studies, pre-post or before-after studies without control group, and cohort studies will be excluded. |
| PICOS= Population, Intervention, Comparison, Outcomes, and Study designs | |

| **Table 2.** : Preliminary search strategy in Ovid MEDLINE | | |
| --- | --- | --- |
| **Concepts considered** | **Search strategy** | **Results** |
| Referral | "Referral and Consultation"/ or Gatekeeping/ | #1 |
|  | (referr* or decision to refer).tw,kf. | #2 |
|  | (gate keeping or gate keeper* or gatekeeping or gatekeeper* or second opinion* or consultation*).tw,kf. | #3 |
|  | #1 OR #2 OR #3 | #4 |
| Clinical decision support | Decision Support Systems, Clinical/ | #5 |
|  | Clinical Decision Rules/ | #6 |
|  | Decision Trees/ | #7 |
|  | decision support*.tw,kf | #8 |
|  | decision support techniques/ | #9 |
|  | Clinical Decision-Making/ | #10 |
|  | (decision tree* or decision rule* or decision aid or decision aids or decision analysis or decision analyses or decision model*).tw,kf | #11 |
|  | (clinical prediction or clinical rule*).tw,kf. | #12 |
|  | **OR/5-12** | #13 |
| Chronic pain | exp Pain/ or exp Migraine Disorders/ | #14 |
|  | (pain or headache* or migrane* or fibromyalgia or neuralgia).tw,kf. | #15 |
|  | #14 OR #15 | #16 |
| Combination of concepts for final search | #**4 AND** #**13 AND** #**16** | #17 |
